# Eco-friendly spectrophotometric quantification of the potential combination of mirabegron and tamsulosin

**DOI:** 10.1038/s41598-025-06157-9

**Published:** 2025-06-23

**Authors:** Omar M El-Abassy, Hanaa Saleh, Islam M. Darwish, Eman A. Bahgat

**Affiliations:** 1 Pharmaceutical Chemistry Department, Faculty of Pharmacy, Egyptian Russian University, Badr City, 11829 Cairo Egypt; 2 Pharmaceutical Analytical Chemistry Department, Faculty of Pharmacy, Zagazig University, Zagazig, 44519 Egypt; 3 Egyptian Drug Authority, Giza, 12622 Egypt

**Keywords:** Mirabegron, Tamsulosin, Ratio difference, Dual wavelength, Derivative ratio, Chemistry, Analytical chemistry, Green chemistry

## Abstract

Recently, men with overactive bladder have been prescribed mirabegron and tamsulosin for the treatment of benign prostatic hyperplasia. Highly efficient and environmentally sustainable spectrophotometric methods have been developed for the accurate determination of mirabegron and tamsulosin in their pure forms as well as within pharmaceutical formulations. This study presents three effective and simple spectrophotometric methods for the simultaneous quantification of mirabegron and tamsulosin. The current protocols have demonstrated validation for linearity across concentration ranges of 3–20 µg/mL for mirabegron and 2–40 µg/mL for tamsulosin, utilizing dual wavelength, ratio difference, and derivative ratio techniques. The coefficients of determination exceeded 0.999. The validation of these methodologies was conducted in accordance with the guidelines set forth by the International council for Harmonization (ICH). Quality control laboratories may utilize existing techniques to identify the binary combination because of their high accuracy and cheap cost. The evaluation of the environmental sustainability of the established approaches was conducted using AGREE, GAPI, MOGAPI and whiteness revealing their notable eco-friendliness. The proposed method was deemed practical after the evaluation carried out with the Blue Applicability Grade Index (BAGI) assessment.

**Supplementary Information:**

The online version contains supplementary material available at 10.1038/s41598-025-06157-9.

## Introduction

Benign prostatic hyperplasia (BPH) and overactive bladder syndrome (OAB) are prevalent conditions that often lead to lower urinary tract symptoms (LUTS) and diminished quality of life, particularly among the elderly^1–3^. This medical issue is prevalent among adults aged 40 and older, though it can also impact children and adolescents^4^. A research investigation overseen in 2011 involving 10,000 individuals from Europe found that approximately 36% of males and 43% of females aged 40 and older displayed symptoms consistent with OAB^5^. According to the National Overactive Bladder Evaluation (NOBLE) program, nearly 33 million Americans are affected by (OAB). The OAB plays a crucial role in shaping economic policy. In 2005, a study conducted in the US estimated that the yearly costs associated with individuals suffering from OAB exceeded $12 billion USD. This expenditure encompasses indirect costs, including a temporary decline in productivity^6^. Sometimes, α1-adrenergic receptor blockers (α1B) are used to treat (LUTS) in men who have been diagnosed with (BPH). Even with the (α1B) treatment, symptoms of overactive bladder may persist^7^. The 2015 guideline from the European Urologic Association advises the use of antimuscarinics or beta-3-adrenergic receptor agonists (β3-ARs) in men diagnosed with BPH to address moderate to severe LUTS, particularly those associated with bladder storage dysfunction. In situations where single treatments fail to provide symptom relief, it is essential to implement a combined therapy approach^8^.

**Fig. 1:**
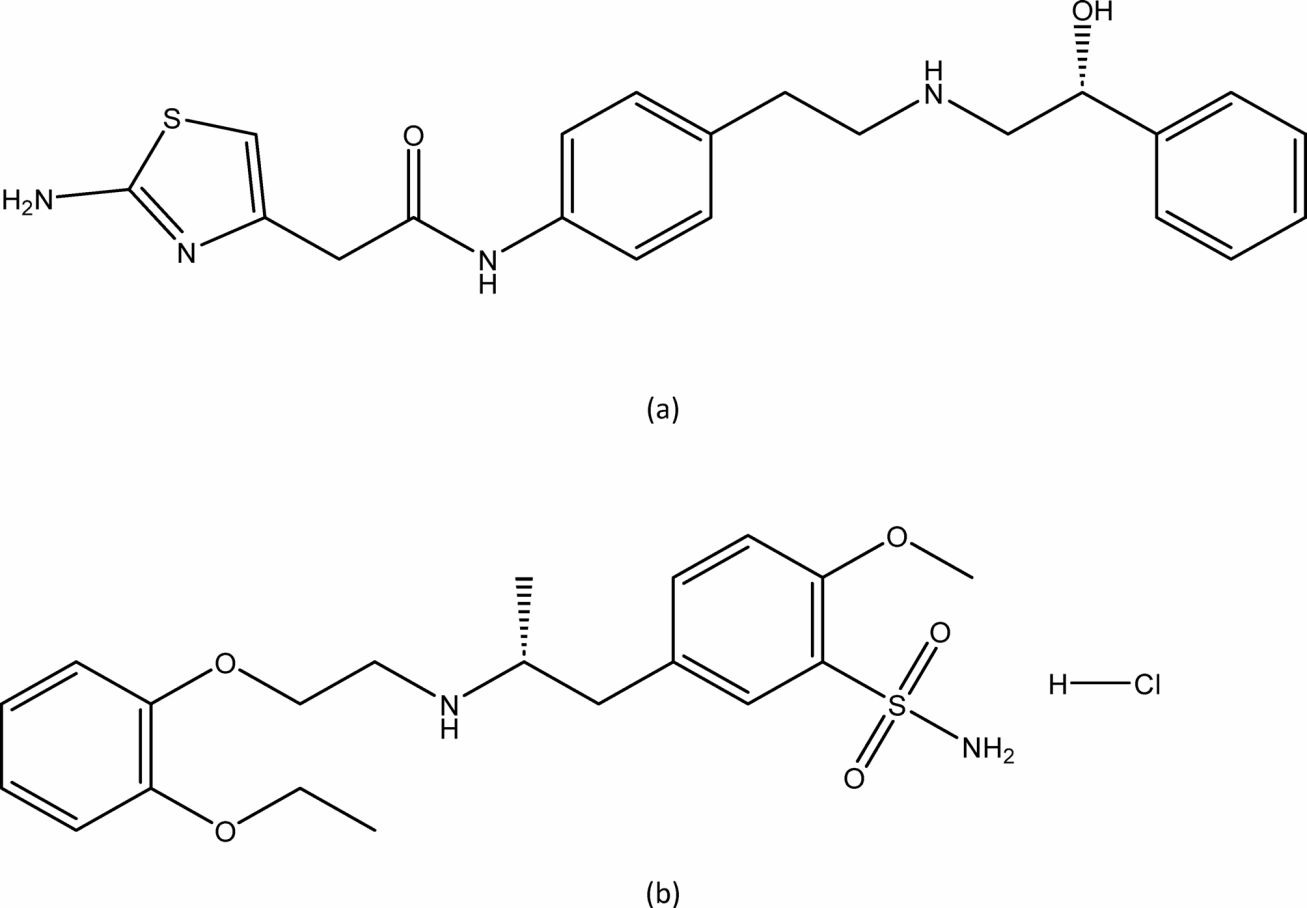
The chemical composition of (**a**) MIR and (**b**) TAM.

Mirabegron (MIR) is a recently approved medicine by the FDA for the treatment of idiopathic overactive bladder^9^. Mirabegron functions as a β_3_ specific receptor agonist, exerting its effects through the relaxation of the detrusor muscle. Regarding the chemical composition of the compound, MIR can be described as (N-[4-[2-[[(2R)-2-hydroxy-2-phenylethyl] amino] ethyl] phenyl] acetamide)^10^ (Fig. 1a).

Tamsulosin (TAM), is α1-adrenoceptor antagonist with the chemical formula 5-[(2R)-2-[2-(2-ethoxyphenoxy) ethylamino]propyl]l.2-methoxybenzene-1-sulfonamide^11^ that is employed in the management of lower urinary tract symptoms linked to benign prostatic hyperplasia (BPH) (Fig. 1b).

Several studies have shown that MIR is an effective and safe adjunct therapy to TAM for alleviating symptoms of OAB resulting from BPH in males, with a low incidence of adverse effects^12^. Current clinical investigations indicate that the combination of medication such as MIR and TAM for overactive bladder (OAB) has shown improvements in symptom relief without further side effects. The combination treatment of TAM and MIR is anticipated to undergo comprehensive investigation as an alternative to traditional pharmaceuticals for patients who are unable to endure the constraints of existing drug regimens or adverse drug reactions^13^. Numerous chromatographic studies have been conducted on MIR detection^14–20^. and estimation of TAM^21–30^, According to the literature, there are HPTLC method^31^ and HPLC method^32^ that have been documented for measuring MIR and TAM simultaneously.

## Materials and methods

### Instrumentation

The UV spectrophotometer was equipped with Jasco spectrum management software. Absorption spectra for test solutions within the wavelength range of 200–400 nm were measured using quartz cells with a diameter of 1 cm.

### Materials, reagents and solvents

Reference standards of TAM (99.75%) and MIR (99.88%) were procured from LGC Standards. The pharmaceutical preparations Betmiga 50 mg^®^ and Tamsul 0.4 mg^®^ were supplied from a community pharmacy. Distilled water and ethanol (spectroscopy grade, EL-Gomhouria Company, Egypt) were the solvents used.

### Preparation of standard stock solutions

Weighing 10 mg of each reference standard and then accurately transfer it into individual volumetric flask 100 ml produced MIR and TAM stock standard solutions with a concentration 100 µg/mL. The compounds were then dissolved and combined in 20 mL of ethanol, then complete the volume to mark with distilled water.

### Preparation of the laboratory prepared mixture

Aliquots of MIR and TAM were transferred from their stock standard solutions into a 10 mL volumetric flask . Then topped to the mark with distilled water and stirred well.

## Experiment design

### Determining linearity ranges and constructing calibration curves

The objective is to achieve concentrations ranging from 3 to 20 µg/mL for MIR and 2 to 40 µg/mL for TAM in Dual Wavelength (DW), Ratio Difference (RD), and Derivative Ratio (DD^1^). The absorption spectra of the resulting solutions for both compounds were documented within the range of 200 to 400 nm, employing mixture of distilled water and ethanol as a blank .

### Dual wavelength spectrophotometric approach (DW)

The curve of calibration was created by measuring the absorbance changes between 240 and 266 nm and plotting these against different concentrations of MIR. Absorbance values at 230 and 262 nm were obtained from TAM spectra to quantify TAM, and the corresponding differences were plotted against TAM concentrations. The charts were utilized to formulate regression equations.

### Ratio difference spectrophotometric approach (RD)

The MIR zero-absorption spectra were acquired, stored, and divided using the 10 µg/mL TAM spectrum as a divisor. Amplitudes of the resultant spectra have been estimated at 220 and 248 nm, and the differences were computed to construct the calibration curve. Zero-absorption spectra of TAM were recorded, saved, and subsequently divided using 7 µg/mL MIR as a divisor. Amplitudes of the resultant spectra have been estimated at 221 nm and 295 nm, and the difference was computed to construct the calibration curve, which was then utilized for regression equation computation.

### First derivative ratio spectrophotometric approach (DD^1^)

In order to create the ratio spectra, the measurements of MIR’s absorption were divided by 10 µg/mL of TAM. One further step was to produce the first derivative of the ratio spectra. Amplitudes of the resultant derivative spectra of MIR have been estimated at 242 nm. To determine TAM in the context of MIR, divide the stored TAM zero-absorption spectra by 7 µg/mL of MIR to obtain the ratio spectra. One further step was to produce the first derivative of the ratio spectra. Amplitudes of the resultant derivative spectra of TAM have been estimated at 302 nm.

### Analysis of laboratory-prepared mixture

The spectra of the laboratory-prepared mixtures was processed as outlined in the experiment design section. The concentrations of each analyte were determined using established regression models.

### Quantification of the pharmaceutical dosage forms

The formulation of Betmiga^®^ involved careful weighing, crushing, and mixing ten tablets. The average weight of a single tablet is 257.5 mg, which is achieved by incorporating 50 mg of MIR into a volumetric flask 100 mL. Twenty Tamslu^®^ capsules contents were carefully weighed, and their average weights were calculated. The contents of the capsule were carefully combined and the content corresponding to a mass of 5 mg TAM was transferred to a volumetric flask 25 mL. The flasks were filled with 30 mL and 15 mL, respectively, utilizing ethanol. The flasks underwent stirring with an ultrasonic shaker for a duration of 15 min. Following the filtration process with a whatman filter, the contents of the flasks were subsequently diluted using distilled water. Aliquots of Betmiga^®^ and Tamsul^®^ filtrates were transferred into a volumetric flask 25 mL and subsequently diluted to the final volume with distilled water to prepare a solution of MIR and TAM.

## Results and discussion

**Fig. 2:**
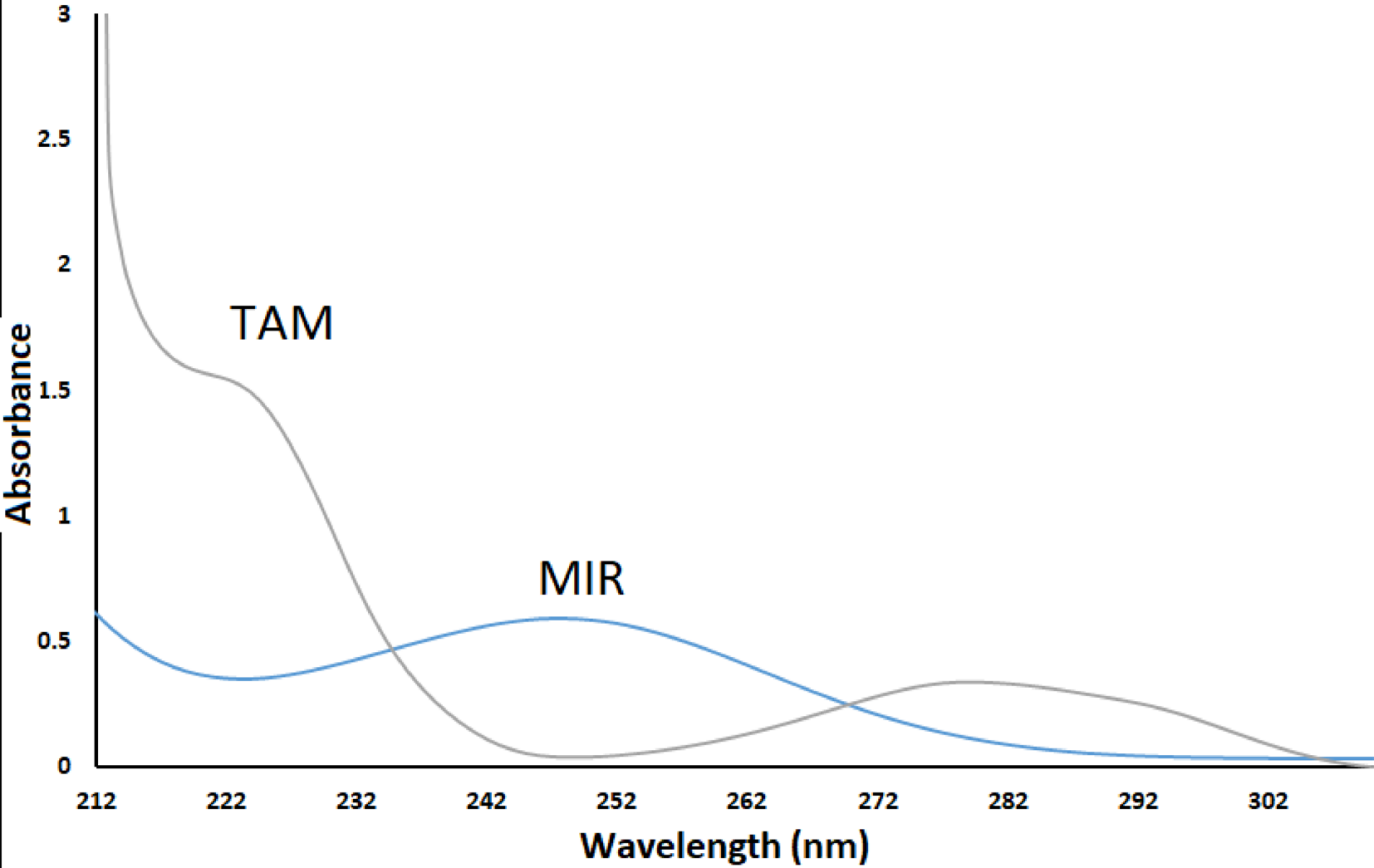
Spectra of zero order for MIR (10 µg/mL) and TAM (30 µg/mL).

The concurrent use of MIR and TAM is noteworthy for its effectiveness in alleviating symptoms of bladder obstruction in older adults. Three distinct, delicate, simple UV-spectrophotometric approaches have been developed for the simultaneous measurement of MIR and TAM in synthetic mixtures and pharmaceutical formulations, ensuring sufficient accuracy outcomes for competent analytical methodology. The proposed UV approach is efficient, economical, eco-friendly, and precise for routine drug analysis. The spectra presented in Fig. 2 demonstrate significant overlap, indicating that MIR and TAM can be calculated using three advanced platforms. These platforms are effective in selectively eliminating interference or overlap in the combined mixing spectra without prior separation.

### Methods optimization

#### Dual wavelength spectrophotometric approach (DW)

**Fig. 3:**
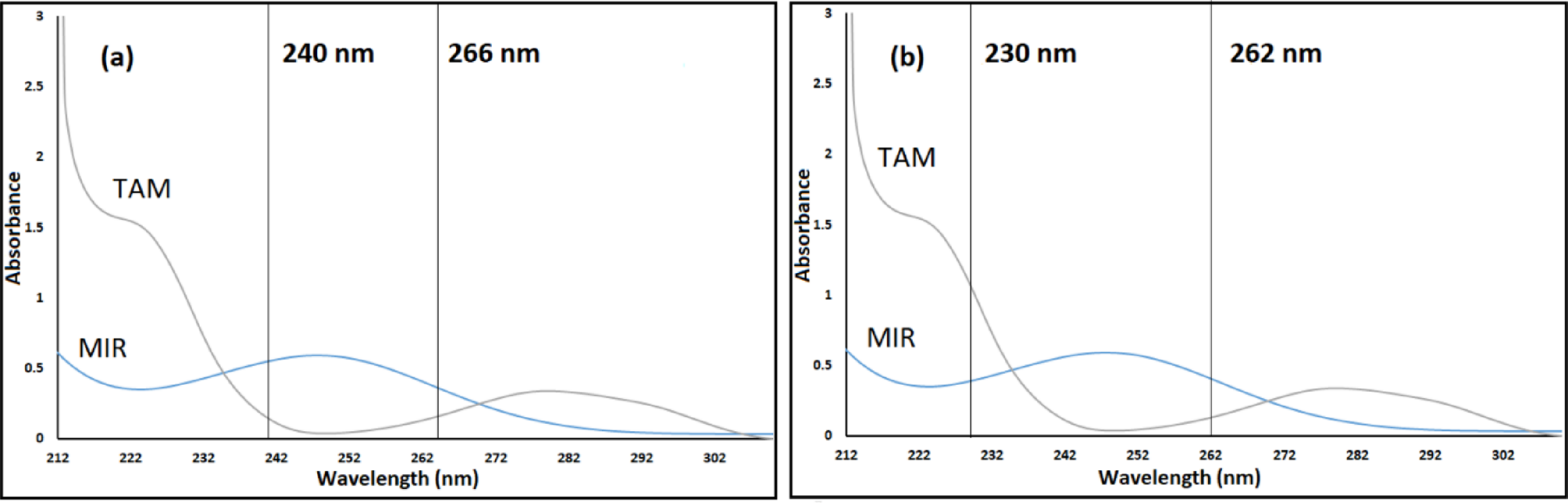
(**a**) Zero absorption spectrum of 10 µg/mL MIR overlaid with 30 µg/mL TAM revealed that 240 nm and 266 nm has the same absorbance in TAM and (**b**) Zero absorption spectrum of 30 µg/mL TAM overlaid with 10 µg/mL MIR revealed that 230 nm and 262 nm has the same absorbance in MIR.

DW effectively reduces interference between MIR and TAM in their mixed formulations due to its simplicity and lack of requirement for specialized software^33^. Additionally, no further data processing is required. To mitigate interference, two wavelengths were chosen from the mixture spectra, ensuring that the absorbance difference between these wavelengths is directly proportional to the concentration of the target compound, while remaining zero for the interfering compound. The wavelengths used for determining MIR and TAM are detailed in the experiment design and depicted in Fig. 3. Regression equations were established to determine MIR and TAM concentrations by plotting the absorbance difference values at specific wavelengths of zero-order spectra against concentration ranges of 3–20 µg/mL and 2–40 µg/mL, respectively.

#### For ratio difference approach (RD)

**Fig. 4:**
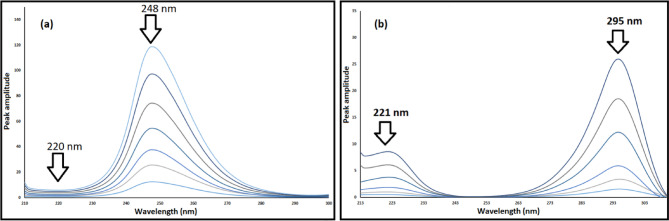
Spectra of the ratios of (**a**) MIR (3–20 µg/mL) utilizing 10 µg/mL of TAM as a divisor and (**b**) TAM (2–40 µg/mL) utilizing 7 µg/mL of MIR as a divisor.

Ratio Difference is a basic computational solution that uses simple mathematical calculations and doesn’t need separation^34^. This technique relies on the appropriate selection of divisors, ensuring minimal noise and maximum sensitivity, alongside the wavelengths utilized for computations to achieve optimal linearity relationships. MIR can be calculated in conjunction with TAM using the RD technique, achieved by dividing it by a TAM divisor of 10 µg/mL. The amplitudes of the resultant ratio spectrum at 220 and 248 nm were measured to eliminate TAM interference, and the difference in amplitude was subsequently calculated. TAM was measured in relation to MIR by using a MIR divisor of 7 µg/mL for the calculation. The amplitudes of the resultant ratio spectrum at 221 and 295 nm were measured to eliminate MIR interference, and the difference in amplitude was subsequently calculated. Regression equations were developed to ascertain MIR and TAM concentrations by graphing absorbance difference values at designated wavelengths against concentration ranges of 3–20 µg/mL and 2–40 µg/mL, respectively. (see Fig. 4).

#### First derivative of the ratio spectra method (DD^1^)

**Fig. 5:**
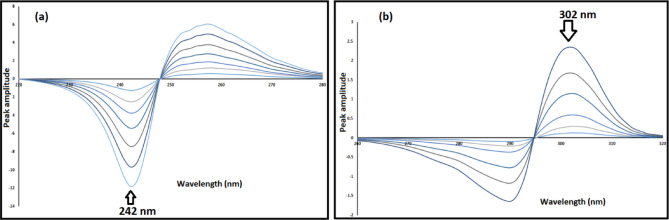
First derivative of ratio spectra of (**a**) MIR (3–20 µg/mL) (**b**) TAM (2–40 µg/mL).

This method relies on the first derivative of ratio spectra, achieved by dividing the zero-order mixture spectra of the analyzed drugs by a specifically selected spectrum of 10 µg/mL TAM to ascertain MIR. Additionally, the zero-order absorption spectra of TAM are divided by a specifically chosen spectrum of 7 µg/mL MIR to determine TAM, after evaluating various concentrations as divisors for both drugs (Fig. 5)^35^.

### Validation of methods

The existing approaches were created in accordance with ICH principles and requirements ^36^.

#### Linearity and range

Seven and six concentrations of MIR and TAM within linearity ranges of 3–20 µg/mL and 2–40 µg/mL, respectively, were used to evaluate the linearity of the proposed spectrophotometric methods (Table 1).

**Table 1:** Spectrophotometric parameters for MIR determination with TAM.

Parameters		DW		RD		DD^1^	
		MIR	TAM	MIR	TAM	MIR	TAM
Range (µg/mL)		3–20	2–40	3–20	2–40	3–20	2–40
Determination coefficient		0.9996	0.9998	0.9994	0.9997	0.9994	0.9998
Slope		0.03	0.02	5.86	0.42	0.61	0.05
Intercept		− 0.03	0.01	− 5.70	0.07	− 0.61	0.008
LOD (µg/mL)		0.36	0.57	0.45	0.61	0.44	0.54
LOQ (µg/mL)		1.09	1.75	1.38	1.87	1.34	1.64
Wavelength		240–266 nm	230–262	220–248 nm	221–295 nm	242 nm	302 nm
Accuracy (Recovery%±SD)		98.90 ± 0.75	100.12 ± 0.68	100.12 ± 0.42	99.97 ± 1.02	99.73 ± 0.38	99.17 ± 0.95
Precision %RSD	Intraday	0.55	0.45	0.65	0.53	0.28	0.94
	Interday	0.87	0.55	0.77	1.26	0.38	1.18

#### Limit of detection (LOD) and limit of quantification (LOQ)

Measurements of detection and quantification were used to assess the sensitivity of the proposed methods. Calculations for LOD and LOQ were based on the following calculations, which relied on using the standard deviation (σ) of the intercept and the slope (S) of the calibration plot given in Table 1. 

**LOD = 3.3 x** σ **/ slope**.

**LOQ = 10 x** σ **/ slope**.

#### Accuracy

Three separate drug concentrations, with each concentration tested three times, were used to assess accuracy. After that, we compared the measured concentrations to the actual concentrations and found the percentage recovery (R%). The results showed that the recovery values were within the permitted range, which proves that the approaches are very accurate (Table 1).

#### Precision

Three distinct concentrations of the proposed drugs, within the linear range, were evaluated by three replicate studies conducted on the same day and again three days later to ascertain the intra-day and inter-day precision of the proposed methods; the findings are shown in Table 1.

#### Selectivity

The approaches’ selectivity was evidenced by the quantitative measurement of MIR and TAM in their laboratory-prepared combinations, as outlined in (Table 2).

**Table 2:** Analysis of MIR and TAM blends made in the lab using the suggested spectrophotometric techniques.

Methods	DW		RD		DD^1^	
Concentration (µg/mL)	Found %					
	MIR	TAM	MIR	TAM	MIR	TAM
MIR: TAM						
15:15	99.00	99.04	100.08	100.18	99.68	99.05
10:20	99.48	100.10	98.12	98.94	98.05	98.24
18:9	100.12	100.59	100.09	100.52	100.24	100.07
20:4	98.80	98.13	101.41	99.88	100.73	99.37
5:30	98.44	101.05	101.74	98.38	101.60	97.92
Mean ± SD	99.17 ± 0.65	99.78 ± 1.18	100.29 ± 1.42	99.58 ± 0.89	100.06 ± 1.32	98.93 ± 0.86

### Pharmaceutical formulations application

The three approaches (DW, RD, DD^1^) were utilized in the application of dosage forms. Analysis of MIR in tablet form and TAM in capsule form demonstrated an average recovery rate of 100.70%, 99.80%, 100.40% and 99.78%,99.86%, 99.62%, correspondingly (Table 3).

**Table 3:** Statistical comparison of tablet results between the proposed and published methods.

Parameters	MIR				TAM			
	Proposed methods			Reported method^31^	Proposed methods			Reported method^31^
	DW	RD	DD^1^		DW	RD	DD^1^	
Mean	100.70	99.80	100.40	99.67	99.78	99.86	99.62	99.84
SD	1.04	1.15	1.02	0.68	1.08	1.09	1.24	0.90
n	5	5	5	3	5	5	5	3
Student’s t-test (2.45)	1.69	0.20	1.21		0.08	0.03	0.29	
F-value (19.25)	2.34	2.86	2.25		1.44	1.47	1.90	

**Fig. 6:**
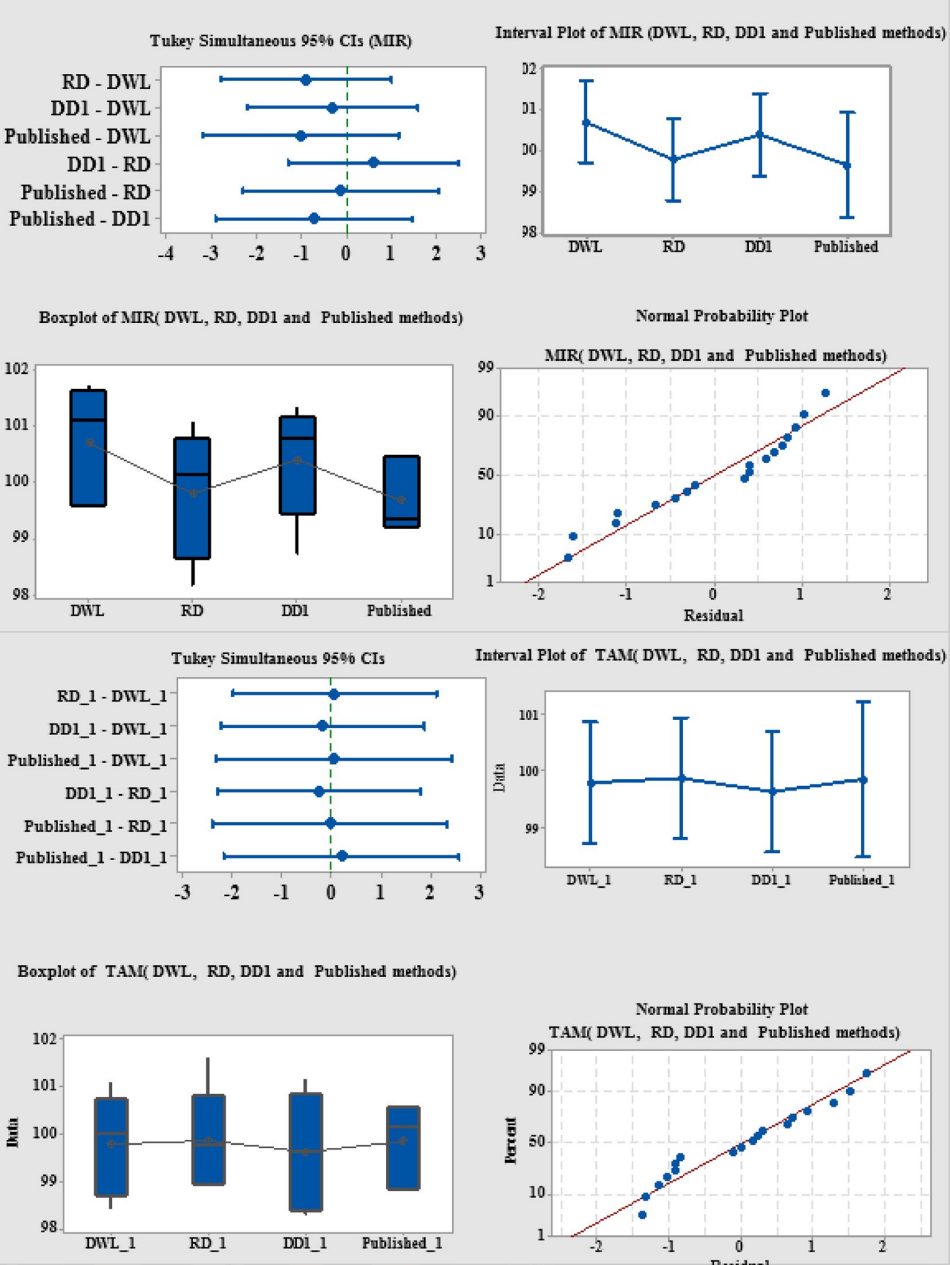
Comparison between proposed methods with each other and with HPTLC published method.

The student’s t-test and the F-test were used to compare the suggested methods with the published method statistically. Table 3 shows that there were no significant differences between the two approaches. Methods were systematically compared against one another and published results through various statistical techniques. The test of interval plot was used^37^. Plots with vertical lines representing confidence intervals imply that the interval’s mean is located near its midpoint. According to the graphs, there are no appreciable variations in the group intervals. (Fig. 6). One useful technique for visualizing data is the boxplot, which illustrates the distribution of data across various sets^38^. The boxplots for the established and published approaches are shown. The central box depicts the interquartile range, this comprises a line representing the median, upper lines indicating higher numbers, and whiskers showing lower values. The boxplot shows the distribution of data within each group (Fig. 6). The normal probability plot offers an additional method for determining the normality of data distribution (Fig. 6). If a straight line connects the majority of data sets, the data follows a normal distribution^37^. Tukey’s simultaneous significant difference test is the most important statistical technique^39^. This tool effectively identifies variations in mean values across different groups. The data interval for each group is illustrated in (Fig. 6) as a horizontal line, with a dot indicating the mean value of the corresponding data group. The overlap of the intervals suggested that the average values of the planned and reported methods were not significantly different.

### Assessment of ecological effect and comparison to reported analytical technique

During the development of analytical procedures, evaluating their ecological impact was essential to ensure a transparent and factual assessment for future comparisons of proposed and reported methodologies. The Green Analytical Procedure Index (GAPI)^40–43^ was utilized to evaluate the environmental sustainability of the analytical methodologies. The environmental implications of each step in analytical measurement, encompassing preparation, equipment use, and waste generation and management, are evaluated by GAPI, which comprises fifteen unique domains illustrated by five pentacle shapes. The characteristics are illustrated on the pentagram, where the colors green, yellow, and red signify minimum, moderate, and significant environmental impact, respectively. The suggested spectrophotometric techniques exhibit numerous benefits in comparison to the previously documented HPTLC chromatographic approach. The considerations encompass the application of eco-friendly solvents, a reduction in solvent consumption during analysis, lower toxicity levels of solvents, decreased energy demands for the spectrophotometric apparatus, minimized waste generation, and solvent degradation **(**Table 4**)**.

**Table 4:** A summary comparing the recommended and published methods.

Parameter	Proposed methods	Published method^*^ [31]
Technique	Spectrophotometric methods (DW, RD, DD^1^)	HPTLC
GAPI		
AGREE		
MOGAPI		
BAGI		

*Separation was done on silica gel F_254_ solvent system methanol-ethyl acetate-ammonia (3:7:0.1, v/v), detection was done at 270 nm*.

Using the GAPI statistic to compare many methodologies does not provide a comprehensive result. The MoGAPI program amalgamates the exact total score of the analytical Eco Scale with the benefits of GAPI’s visual effect. As illustrated in Table 4, the software program was implemented to evaluate the ecological sustainability of the recommended analytical and HPTLC published methods^44^.

AGREE features a clock-shaped diagram segmented into twelve parts, where each part represents one of the twelve principles of Green Analytical Chemistry^45,46^. The proposed and reported HPTLC methods were assessed using the AGREE tool. The comparison presented in Table 4 accurately outlines the proposed methodology against previously reported methods, employing real data for analysis. The two methodologies demonstrate a red zone (3) on the AGREE scale. A significant issue has arisen due to offline sampling and the transportation of samples to quality control laboratories, stemming from the division between pharmaceutical manufacturing and quality control sites. The proposed spectrophotometric methods exhibit several advantages compared to conventional chromatographic techniques, including fewer stages, lower waste volume, reduced energy consumption of the spectrophotometric device, elimination of toxic solvents, and decreased dependence on organic solvents (Table 4).

The proposal introduces an innovative metric, the Blue Applicability Grade Index (BAGI), aimed at evaluating the feasibility of an analytical method^47–49^. The BAGI framework, when utilized in conjunction with recognized green metrics, elucidates the practical dimensions of White Analytical Chemistry. The color gradient of the pictogram reflects the degree of alignment with the established criteria. For the strategy to be deemed “practical,” it must attain a score of 60 or above. The proposed methodology demonstrates superior performance relative to the previously mentioned HPTLC method, as illustrated in Table 4.

The proposed method was compared to two established methods, including HPTLC and HPLC approaches using the RGB 12 model^50^, both of them had been published before. It is among the most recent multi-criteria evaluation method available and is in line with White Analytical Chemistry’s (WAC) principles. It considers more than only the influence on the environment. It is composed of 12 algorithms divided into three colored groups and is based on a more flexible approach. Numerous evaluation indicators for important aspects of the analytical process make up each colored category. **Table ****S1** displays the findings of this comprehensive whiteness evaluation.

## Conclusion

The suggested approaches provide spectrophotometric procedures that are simple, sensitive, and accurate for MIR and TAM. In recent clinical investigations, the combined therapy of MIR and TAM for over active bladder (OAB) has demonstrated advantages in terms of symptom alleviation without creating additional side effects. Utilizing the spectrophotometric methods that have been provided, it is possible to do an analysis on the overlapping spectra of MIR and TAM, in addition to more complex combinations, utilizing a wide range of tools. The proposed and reported spectrophotometric techniques did not vary statistically significantly, according to a statistical analysis using the t-test and the F-test. In order to assess the impact that these methods have on the environment and determine whether or not they are feasible, the GAPI index, MOGAPI AGREE calculator, BAGI and whiteness evaluation were utilized.

## Electronic supplementary material

Below is the link to the electronic supplementary material.

Supplementary Material 1

## Data Availability

All data generated or analyzed during this study are included in this published article.
