# Supplementary material for: Eco-friendly spectrophotometric quantification of the potential combination of mirabegron and tamsulosin
Source: Sci Rep. 2025 Jun 23;15:20252. doi: 10.1038/s41598-025-06157-9 (PMC12185759; doi:10.1038/s41598-025-06157-9)
Supplement: Supplementary file 1 — Supplementary Material 1 [file 41598_2025_6157_MOESM1_ESM.docx]

| Published method [32] | Published method [31] | Proposed methods |
| --- | --- | --- |
| 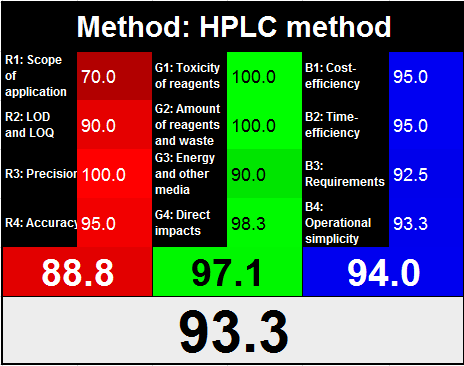 | 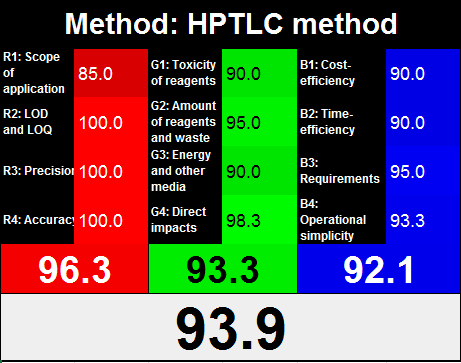 | 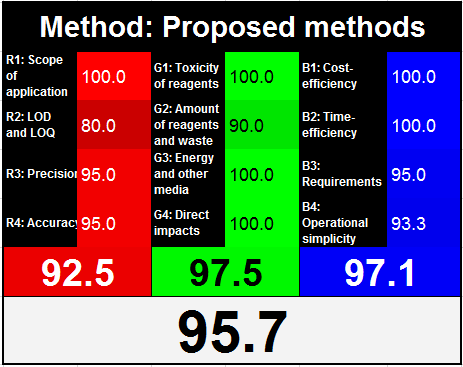 |
|  |  |  |

**Table S1: Whiteness comparison between proposed and published methods**
